# Discovery of Graphene‐Water Membrane Structure: Toward High‐Quality Graphene Process

**DOI:** 10.1002/advs.202201336

**Published:** 2022-07-18

**Authors:** Aisha Okmi, Xuemei Xiao, Yue Zhang, Rui He, Olugbenga Olunloyo, Sumner B. Harris, Tara Jabegu, Ningxin Li, Diren Maraba, Yasmeen Sherif, Ondrej Dyck, Ivan Vlassiouk, Kai Xiao, Pei Dong, Baoxing Xu, Sidong Lei

**Affiliations:** ^1^ Department of Physics and Astronomy Georgia State University Atlanta GA 30303 USA; ^2^ Department of Physics Jazan University Jazan 45142 Saudi Arabia; ^3^ Department of Mechanical and Aerospace Engineering University of Virginia Charlottesville VA 22904 USA; ^4^ Department of Mechanical Engineering George Mason University Fairfax, VA 22030 USA; ^5^ Department of Physics and Astronomy University of Tennessee Knoxville TN 37996 USA; ^6^ Center for Nanophase Materials Sciences (CNMS) Oak Ridge National Lab Oak Ridge TN 37830 USA

**Keywords:** graphene, polymer‐free transfer, surface tension, ultra‐flatness

## Abstract

It is widely accepted that solid‐state membranes are indispensable media for the graphene process, particularly transfer procedures. But these membranes inevitably bring contaminations and residues to the transferred graphene and consequently compromise the material quality. This study reports a newly observed free‐standing graphene‐water membrane structure, which replaces the conventional solid‐state supporting media with liquid film to sustain the graphene integrity and continuity. Experimental observation, theoretical model, and molecular dynamics simulations consistently indicate that the high surface tension of pure water and its large contact angle with graphene are essential factors for forming such a membrane structure. More interestingly, water surface tension ensures the flatness of graphene layers and renders high transfer quality on many types of target substrates. This report enriches the understanding of the interactions on reduced dimensional material while rendering an alternative approach for scalable layered material processing with ensured quality for advanced manufacturing.

## Graphical abstract

A study of a newly discovered graphene‐water membrane is accomplished. It provides a promising technique for high‐quality graphene process with ultra‐high flatness, and free of defects, leveraging the high surface tension of pure water. These advantages enable high‐performance device fabrication for electronic and biological research.

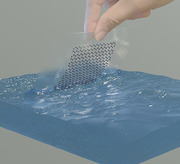

## 1 Introduction

Solid‐state membranes, including polymethyl methacrylate (PMMA),^[^ ^1^ ^]^ paraffin,^[^ ^2^ ^]^ and other solvable organic compound with good membrane‐forming capability, have long been used for the graphene process. These membranes provide outstanding operational flexibility for the transfer process, but inevitably lead to concerns about mechanical deformation, contaminations, and trapped interfacial residues that impact the performances of graphene‐based devices.^[^ ^3^ ^]^ On the other hand, graphene process assisted with liquid membranes has rarely been explored or reported before, due to the assumption that liquid surface tension could potentially damage the graphene integrity,^[^ ^4^ ^]^ or it is impossible to establish free‐standing and stable graphene‐liquid membrane structures.

Here, we report a newly observed graphene‐water membrane (GWM) structure, which refreshes our understanding of graphene‐liquid interaction, meanwhile rendering a new material process procedure. We thoroughly investigate the formation mechanism of the as‐observed GWM, particularly the roles of surface tension and contact angle playing, and discover that the GWM enables a new graphene transfer method that directly renders free‐suspended layer or on‐substrate film with improved flatness, thanks to the high water surface tension, which has long been misinterpreted to jeopardize the graphene transfer process. Further, the GWM transfer eliminates residuals induced by conventional solid‐state membranes, inspiring high‐quality and contamination‐free graphene process pathways toward the subsequent development of novel graphene‐based electronics,^[^ ^5^ ^]^ quantum devices,^[^ ^6^ ^]^ micro‐electromechanical systems,^[^ ^7^ ^]^ and flexible biosensors.^[^ ^8^ ^]^

## 2 Experimental Implementation of GWM

**Figure 1:**
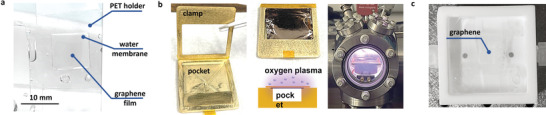
a) Free‐standing GWM held by a PET frame. b) Home‐designed fixture for graphene plasma removal. Copper foil with CVD graphene grown on both sides is clamped on the fixture, with one side exposed to radio‐frequency plasma, while the other side is electrically shielded by the cavity formed with the copper foil and the pocket on the metal fixture. The shielded copper side has no physical contact with the surroundings, ensuring the graphene integrity. c) PTFE reactor with source and drain designed for copper etching and liquid replacement).

**Figure** **1**a shows the optical image of the free‐standing GWM structure observed in our experiment. The successful formation of the GWM requires several precautionary measures to ensure graphene quality and integrity. The graphene employed in our study is synthesized on copper foils in a regular chemical vapor deposition (CVD) system^[^ ^9^ ^]^ with a leakage rate lower than 10^–9^ bar cm^3^ s^−1^ to minimize defects induced by oxygen during the growth. (Figure S1, Supporting Information shows the defects developed when the leakage rate is higher than >10^–7^ bar cm^3^ s^−1^) Following the growth, we preserve the graphene on one side of the copper foil, while etching the layer deposited on the other side with argon plasma. In this process, instead of protecting the desired surface with polymer coating, we designed a plasma treatment fixture, as demonstrated in Figure 1b, to shield the argon plasma for protection. The fixture consists of a top bronze clamp and a bottom copper platform with a pocket. This pocket and clamped copper foil form a Faraday cage to fully block the argon plasma from bombarding the reserved graphene layer; meanwhile, it eliminates physical contact with the delicate graphene layer and ensures its integrity. Additionally, we use argon instead of oxygen plasma for the etching because ozone generated in the oxygen plasma could potentially diffuse into the pocket and oxidize the graphene layer. These efforts ensure the graphene quality and integrity to the maximum extent for the following study on the GWM and the new transfer technique.

With the preserved graphene layer facing up, the copper foil is relocated into a specially designed reactor (Figure S2a, Supporting Information), filled with a 0.1 mol L^−1^ ammonium persulfate ((NH_4_)_2_S_2_O_8_) water solution for copper etching for 3 h, leaving monolayer graphene on the etchant surface. Due to the high surface tension of water (72 mN m^−1^), the thin copper film and the finally released graphene float freely on the surface and are confined by a polyethylene terephthalate (PET) frame with a 1 × 1 cm^2^ opening in the center. Subsequently, we exchanged the etchant with deionized (DI) water and rinsed the monolayer graphene several times to remove all ions, including Cu^2+^ generated during the etching step illustrated in Figure S2b, Supporting Information. To avoid liquid turbulence or fluctuation during the exchange and ensure the integrity of the graphene layer, we designed inlet and outlet ports on the bottom of the reactor, through which the liquid is drained and injected at the same rate. Figure S3, Supporting Information shows the free‐floating graphene before and after the water exchange. A widely accepted viewpoint is that large areas of free‐standing CVD graphene can be easily destroyed by pure water because of its high surface tension.^[^ ^4^ ^]^ In contrast, we did not observe any deformations generated through the entire process, suggesting CVD graphene can stand the high surface tension of DI water as long as the graphene quality is carefully preserved. Otherwise, defects developed during growth and physical damage due to improper handling indeed impair the graphene layer, bringing cracks, wrinkles, shrinking, etc., as shown in Figure S4, Supporting Information.

**Figure 2:**
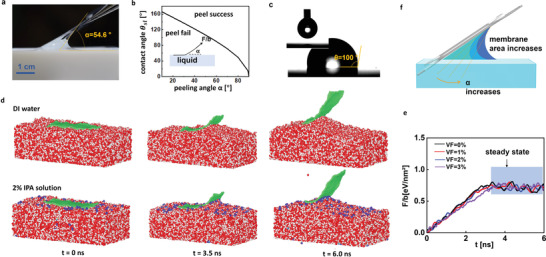
a) Optical image of the graphene peeling process with a peeling angle of 54.6° when pure DI water is employed. b) Criteria of success peeling. The required minimum peeling angle increases and the liquid contact angle decreases (i.e., the liquid becomes more hydrophilic). When the configuration falls in the successful range, the graphene‐water membrane can readily form and be isolated from the liquid surface. If in the failure zone, the membrane breaks. Inset: the definition of peeling angle. c) Contact angle measurement of water on a graphene surface. d) Molecular dynamics simulation of the graphene peeling process. The figures illustrate the frames of the dynamics at 0, 3.5, and 6 s after the peeling starts. The peeling angle is set at 57.5° to ensure a successful peeling in the simulation. e) Simulated evolution of the peeling force as a function of time and IPA concentration. f). A smaller contact angle (induced by higher IPA concentration) requires a larger peeling angle and thus, increases the surface area of the liquid membrane, increasing the risk of membrane breaking and peeling failure.

When a graphene layer is successfully isolated and freely floats on the water surface, as demonstrated in Figure 1c, we can readily peel the free‐floating graphene layer off the water surface by lifting the PET frame from the reactor. This peeling process has been recorded in the optical image shown in **Figure** **2**a. During this step, a water film bridges the graphene layer to the inner edges of the PET frame and pulls it away from the liquid surface. The film buffers the fragile graphene from the PET frame, preventing the sharp edges from piercing the atomic layer, while still distributing the necessary force for peeling. After the entire graphene layer is levered away from the water surface, the GWM shown in Figure 1a emerges.

A potential concern of the GWM is whether the large surface tension can tear the graphene layer after it is lifted from the water surface. To determine this, we performed the following analysis. The Young's modulus (*E*) of monolayer graphene is in the range from 1.05 to 1.1 TPa.^[^ ^10^ ^]^ Considering a graphene thickness (*t*) of 0.33 nm,^[^ ^11^ ^]^ we can calculate the strain (*ε*) on monolayer graphene induced by the water surface tension (*γ*_water_) via the equation:^[^ ^12^ ^]^

(1) $$\varepsilon \;=\frac{2\gamma_{\mathrm{water}}}{\mathrm{Et}}\;$$

Equation (1) shows that the surface tension only results in a strain of 0.02%, far below the fracture strain of graphene. However, if the microscale defects, predominantly if pores or cracks are present, they can rapidly grow under strain and cause brittle fractures to the polycrystal CVD graphene. Thus, the formation and stability of the GWM strongly depend on the quality of the graphene layer rather than the water surface tension.

## 3 Theoretical Modeling of GWM

To understand the peeling process, we established a mechanical peeling model considering two dominating factors that determine a successful peeling: the driving force provided by the water film that bridges the graphene and the inner edges of the PET frame and helps peel off graphene, and the adhesion between the graphene layer and liquid surface to be overcome during peeling. The driving force per unit width (*D*) is provided by the surface tension (*γ*_*l*_) of water in the form of *D* = 2*γ*_*l*_. The force per unit width (*P*) required to peel the graphene can be derived from the energy balance between the work done by the peeling force and the variation of graphene/liquid interfacial energy,^[^ ^13^ ^]^

(2) $$P\;=\frac{\gamma_{l}\times \left(1+\cos\theta_{sl}\right)}{1-\cos\alpha }\;$$

where *θ*_*sl*_ denotes the contact angle between graphene and water, and *α* labels the peeling angle as defined in Figure 2b inset. For a successful peel, we will have *D* ≥ *P*, i.e.,

(3) $$\alpha \geq \arccos\left[\frac{1-\cos\theta_{sl}}{2}\right]$$

For DI water on graphene, we experimentally determined the contact angle of 100° (Figure 2c) by using a goniometer (DataPhysics OCA 15EC), which takes place within the reported range of 95 ≈ 100°.^[^ ^14^ ^]^ Theoretical prediction resulting from Equation (3) shows a minimum peeling angle of 54.1° for a successful peeling of graphene from the water surface. This is consistent with the experimental observation of 54.6° shown in Figure 2a. Figure 2b summarizes the diagram for picking up the graphene from a liquid surface. A smaller contact angle associated with stronger hydrophilicity between graphene and liquid will require a more powerful driving force and thus a larger peeling angle for a successful graphene lifting.

Additionally, we perform molecular dynamics (MD) simulations on the entire peeling process. A graphene film with 4 nm x 10 nm was placed on the surface of the liquid and peeled by applying a mechanical force with the peeling angle of 57.5° at a velocity of 1 nm ns^−1^. Figure 2d shows the simulation snapshots of peeling graphene from water at 0, 3.5, and 6.0 ns, suggesting a neat peeling of graphene film without liquid molecular residues on the peeled graphene. Figure 2e further plots the evolution of the peeling force as a function of peeling time and implies an equilibrium has been achieved 4 ns after the peeling began. The peeling force at the steady‐state is 115.5 mN m^−1^ when pure water is employed, agreeing with the theoretical prediction of 128.6 mN m^−1^ by Equation (3) with water surface tension of 72 mN m^−1^.^[^ ^15^ ^]^

Besides the DI water, we also consider the peeling process when IPA is added to the water to comprehend the effects of lower surface tension and contact angle. Experimentally, we successfully peeled the graphene layers out of 1% and 2% IPA solution with peeling angles of 55.5° and 57.0°, respectively, which further confirms the theoretical prediction of the “successful peel” region shown in Figure 2c, (Refer to Figure S5, Supporting Information). MD simulations also indicate that the peeled graphene remains clean without residual IPA molecules, similar to peeling from the water surface, as shown in Figure 2d. As the IPA concentrate increases to 3%, the liquid (water with 3% IPA) membrane breaks before the graphene is fully peeled off. Consequently, the entire process fails because a higher IPA concentration decreases the contact angle and necessitates a larger peeling angle, which in turn stretches the liquid membrane until it breaks before the whole graphene layer is lifted from the liquid surface, as illustrated in Figure 2f. Quantitively, our MD simulation finds that a 3% IPA solution leads to a 94.5° contact angle and requires at least a 57.4° peeling angle, which will break the liquid membrane between frame and graphene and fail to pick up the graphene. The peeling force remains approximately the same as a small amount of IPA barely influences the contact angle of the water, as suggested by the theoretical predictions shown in Figure S6, Supporting Information.

The discussed experimental and theoretical analyses indicate that high‐quality graphene can readily stand the high surface tension of water. Interestingly, leveraging the large contact angle between graphene and water (i.e., high hydrophobicity), a stable GWM forms when free‐floating graphene is peeled out of the water surface with a frame structure. This observation refreshes our understanding of graphene‐water interaction and exhibits a new hybrid membrane structure that inspires us with a new large‐scale polymer‐free graphene process technique with excellent outcomes.

## 4 GMW‐Enabled One‐Step Graphene Suspension with No Polymer Residues

**Figure 3:**
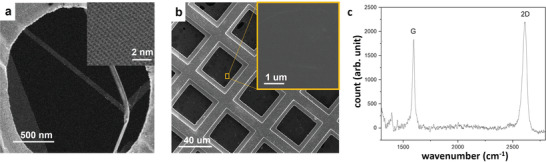
a) STEM image of graphene transferred with GWM. The low‐resolution image shows clean graphene surface, and the high‐resolution image (inset) clearly distinguish carbon atoms. b) SEM image of free‐suspended graphene directly produced by the GWM, and the magnified image (inset) shows no contamination. c) Raman spectrum of the free‐suspended graphene.

One obvious benefit of this GWM is the ensured cleanness of the graphene layer by eliminating the usage of PMMA or other supporting media for processing. Thus, small organic molecules dispersed in the air turn out to be the only contamination source, which can be readily removed by afterward annealing. Indeed, the transition electron microscopy (TEM) image shown in **Figure** **3**b reveals a very clean graphene surface rendered by the GWM, and the scanning TEM (STEM) can clearly distinguish individual carbon atoms.

Besides the ultra‐cleanness, another striking significance of the GMW distinct from other process methods is a one‐step and direct graphene suspension without any assistance of supporting media or supercritical drying employed in earlier studies.^[^ ^16^, ^17^ ^]^ Figure 3c shows the scanning electron microscopy image of a graphene suspension directly obtained by transferring a GMW on a mesh TEM grid with 40 × 40 µm^2^ openings. The inset magnification exhibits the uniform and flawless graphene layer. This one‐step graphene suspension can be attributed to the high graphene quality, and more importantly, the reduced amount of water adhered to the graphene surface when the GMW forms, as indicated by our molecular dynamics simulation that no water adheres to the graphene layer (Figure 2d and Figure S7, Supporting Information) when the GWM is peeled from the water surface. Therefore, the GWM can drastically simplify the procedure for large‐scale suspended graphene and other 2D material structure constructions.

Based on the free‐suspended graphene, we also performed Raman characterization, as shown in Figure 3c, which shows no signal of amorphous carbon.^[^ ^18^ ^]^ The reduced 2D/G peak ratio is well‐known for free‐standing graphene that is charge neutral,^[^ ^19^, ^20^ ^]^ further suggesting the contamination‐free surface of the graphene produced by the GWM.

## 5 GWM‐Enabled High‐Quality Graphene Transfer On Substrates

**Figure 4:**
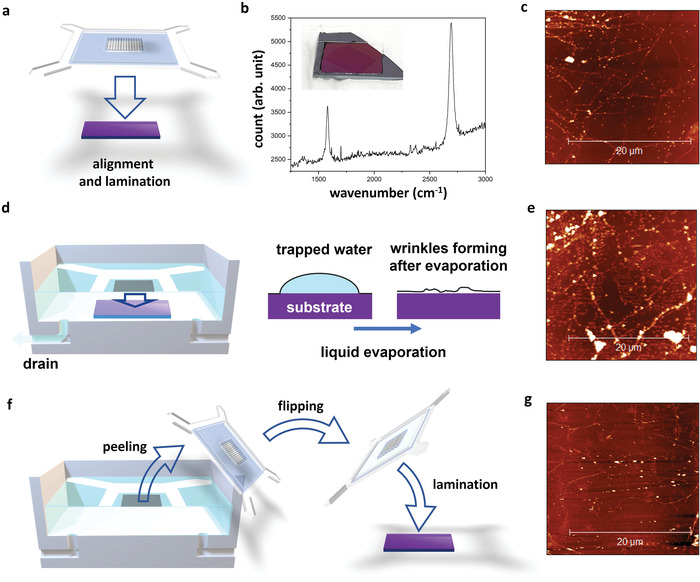
a) Graphene‐wafer membrane for transfer. b) Raman spectrum of the as‐transferred graphene. Inset: Optical image of the as‐transferred graphene. c) AFM image of graphene transferred on Si/SiO_2_ wafer with graphene‐water membrane. d) Conventional graphene transfer method with target‐substrate merged into liquid. The liquid is drawn out of the reactor to lower graphene and laminate it onto the substrate. Because there is liquid trapped on the graphene‐substrate interface, wrinkle develops after the liquid evaporates. e) AFM image of graphene transferred onto Si/SiO_2_ wafer with the conventional polymer‐free procedure. f) Graphene transfer procedure with flipped graphene‐water membrane to further eliminate water trapping on interfaces. g) AFM image of the flipping transfer with better flatness and transfer quality.

The GWM can also be leveraged for graphene transfer onto many types of substrates. Briefly, we can readily pick up the free‐floating graphene layer from the water surface with the PET retainer, then align and laminate it onto the target substrate, as shown in **Figure** **4**a. Here, we employ Si wafers with a 300 nm SiO_2_ layer as the substrates to perform the transfer experiment. They are cleaned with piranha solution (H_2_SO_4_: H_2_O_2_ (37%) = 4:1) to remove organic residues thoroughly. Figure 4b inset shows the optical image of high‐quality large‐area graphene transfer performed with the above procedure, indicating that the sample's integrity is fully preserved without visually detectable cracking or wrinkling. The Raman spectroscopy measurement (Figure 4b) clearly distinguishes the sharp G and 2D peaks with an intensity ratio of 1:2, whereas the D peak is very weak. These observations indicate the monolayer graphene with excellent quality.^[^ ^10^, ^21^ ^]^ To further confirm the microscopic structure and flatness, we conducted an atomic force microscopy (AFM) study on the graphene transferred with our new method and confirmed a low morphology roughness (i.e., the root mean square (RMS) of the surface height) of 1.5 nm, as demonstrated in Figure 4c. Graphene grain boundaries can also be clearly distinguished on the AFM image. In comparison, the transfer following the previously reported polymer‐free method (Figure 4d) results in the roughness of 3.4 nm, as shown in Figure 4e.

One primary reason for this drastic improvement can be attributed to the water film, which bridges the PET frame and graphene layer while stretching and flattening the atomic layer with its surface tension, as explained by Equation (1). Another reason for the much higher transfer quality results from less liquid being trapped on the graphene‐substrate interface in our procedure than in the conventional polymer‐free transfer technique, as confirmed by MD simulations in Figure 2d. In the previously reported polymer‐free transfer methods,^[^ ^2^, ^4^, ^22^ ^]^ target substrates are typically fully merged into the transfer liquid (water or IPA mixture, as illustrated in Figure 4d. Then, the liquid is drained out of the container to lower the graphene layer until it is in contact with the target. This procedure inevitability creates multiple pockets of trapped liquid on the graphene‐substrate interface. Even after the trapped water eventually evaporates, folding, wrinkling, or other deformations persist, as illustrated in Figure 4d, and lead to the as‐observed RMS roughness of 3.4 nm. This problem is detrimental to the afterward device fabrication and test. Nevertheless, our approach can be effectively addressed in such a manner that the hydrophobic nature of graphene repels most of the water accumulated underneath the layer unless minor residuals are anchored by hydrophilic functional groups, including hydroxyl,^[^ ^14^ ^]^ due to unintentional graphene oxidation. Undeniably, our MD simulation further confirms that water molecules do not adhere to the graphene layer being peeled off, as shown in Figure S7, Supporting Information, even if IPA is added and lowers the graphene contact angle. To eliminate the minor interfacial residues or contaminations, we can flip the entire PET frame with a graphene‐water membrane and laminate the “dry” surface onto the substrates. Figure 4f showcases the workflow of this flip‐transfer method. By doing so, we find that the roughness of the resulting transfer is improved to 0.7 nm (Figure 4g), which is close to the intrinsic roughness of SiO_2_ (0.4 nm) used in our study. The improvement in graphene flatness drastically enhances its electronic performance. For example, compared with the conventional polymer‐free transfer method shown in Figure 4d, our GWM‐transfer doubles the FET mobility, as illustrated in Figure S8, Supporting Information, suggesting it can serve as a promised method for high‐quality electronic device fabrication. It is worth mentioning that shadow masks were employed for graphene channel etching and electrode deposition to avoid the usage of photoresist, which could bring residues and uncertainties that interfere with the comparison. As a tradeoff, our FET channels have a relatively large size of 1 mm × 4 mm, which makes the scattering on graphene grain boundary and substrate interfaces more signification, as such lowering the absolute mobility value. On the other hand, this large‐scale design averages the local mobility fluctuation induced by the above factors, and makes our conclusion of mobility enhancement more statistically convincing, compared with micrometer‐scale devices. Additionally, the Dirac cone feature can be clearly distinguished even in these large‐scale FET devices, confirming the high quality of our transfer methods.

**Figure 5:**
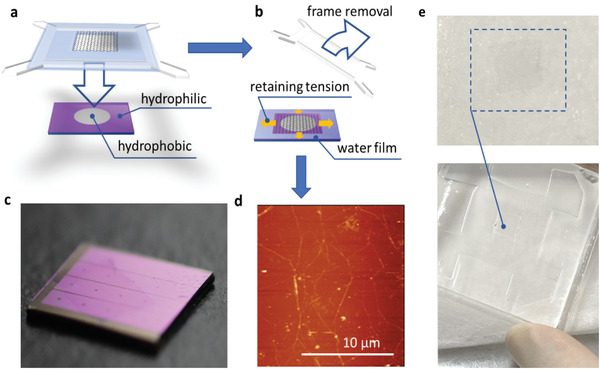
a) Graphene‐water membrane transfer onto a hydrophobic substrate with the assistant of a hydrophilic frame. b) The hydrophilic frame holds the water and retains the tension that flattens the graphene layer until the graphene layer is laminated firmly onto the substrate. c) Optical image of graphene transferred onto silicon with SiO_2_ frame severing as a hydrophilic frame. d) AFM image of as‐transferred graphene on the silicon surface is shown in (c). e) The graphene layer is transferred on the hydrogel (1 g of agar and 0.2 g gelatin dissolved in 100 ml DI water).

Aside from the interpretation of the surface tension effect on the peeling processes, we also identified the substrate factors, particularly the hydrophilicity effect on the success and quality of our new transfer method. Accordingly, we notice that a hydrophilic substrate can sufficiently sustain the integrity of the water membrane and thus the graphene layer. The water keeps tensioning the graphene layer until it fully volatilizes and leaves a highly flat morphology. However, if the substrate is highly hydrophobic, the water film bridging collapses and drags the entire graphene layer to one side of the PET frame instantaneously when it touches the surface, failing the transfer, as illustrated in **Figure** **5**a. To overcome this problem and enable the transfer onto a hydrophobic substrate, a hydrophilic frame surrounding the hydrophobic area is introduced to stabilize the graphene‐water film and sustain the tension until the transfer is finished, as demonstrated in Figure 5b. For example, a SiO_2_ frame was patterned on Si substrate (Figure 5c) through a thermal oxidation process for transferred and form Si‐graphene junction. The entire substrate was treated with hydrogen fluoride acid (5% aqueous solution) before the transfer to passivate the silicon area with hydrogen atoms and produce a hydrophobic surface, whereas the SiO_2_ frame remains hydrophilic. With the assistance of this frame, we successfully transferred graphene onto the Si and obtained ultra‐high flatness, as verified by the AFM image in Figure 5d. Other hybrid structures can also be produced with the same method. Further, Figure 5e illustrates that our newly developed method can effectively transfer graphene to other unconventional substrates, such as hydrogel and other soft‐matters, whereas earlier polymer‐assisted or polymer‐free methods are infeasible because the organic solvent for the afterward cleaning or the full liquid transfer environment can easily damage the hydrogel structure. Therefore, our method also facilitates the fabrication of large‐scale hybrid structures of graphene and soft matters for the applications of biosensors, wearable devices, and many more.^[^ ^5^ ^]^

## 6 Conclusion

We report a new GWM structure and analyze its forming mechanisms and prerequisites from the perspectives of experimental observation, theoretical models, and MD simulations. It is found that the water plays an essential role by providing critical buffering between fragile graphene layers and sharp PET frame for graphene peeling. Also, the large surface tension and contact angle of water deliver sufficient peeling force to isolate graphene layer from the water surface, facilitating the forming of a GWM. Based on this newly observed GWM, we also developed an alternative large graphene transfer method with drastically improved transfer flatness and electronic performances, in part due to the high surface tension of water, which retains the graphene flatness through the entire pickup, alignment, and lamination workflow. These benefits provide an alternative approach for high‐performance device fabrication based on graphene and other low‐dimensional materials.

## 7 Experimental Section

### CVD Growth of Graphene

A homebuilt CVD system had been used to grow the monolayer graphene. It is electropolished 0.025 mm copper foil (CU000358 from Goodfellow) is mainly 80% phosphoric acid (H_3_PO_4_) to achieve the graphene growth later at 1000^°^ C by controlling methane (CH_4_) flowrate at 35 sccm and a mixture of argon and hydrogen (Ar 90%: H_2_ 10%) at 6 sccm.

### Copper Etching

To etch copper, it is dissolved 4.56 g of ammonium persulfate ((NH_4_)_2_S_2_O_8_) (from Sigma‐Aldrich) in 200 ml of DI water to prepare 0.1 M concentration.

### Hydrogel Substrates

were prepared by mixing 1 g of agar powder and 0.2 g of gelatin sheets in 100 ml of boiling DI water. The mix was poured later in a plastic mold and left to solidify for 20 minutes.

### FET Device Fabrication

We fabricated shadow masks by following photolithography methods. Later, the electrode shaped mask to deposit Cr 5 nm Au^−1^ 45 nm using a thermal vapor deposition system (thermal evaporator EDWARDS Auto 306) is used. All the FET devices were annealed after fabrication at 300^°^ C for one hour under vacuum. Finally, it is shaped to transfer graphene to narrow channels (width = 1 mm) using radiofrequency argon plasma and the strips’ shadow masks.

### Characterization

Raman study on suspended and on‐substrated graphene was performed with 785 and 514.4 nm excitations, respectively. AFM scanning of graphene on the Veeco MultiMode AFM system under tapping mode is performed. The measurements of electron mobility on graphene FET devices were done on a homebuilt probe station combined with a source meter unit (SMU, Keithley 2450). SEM was performed on a Zeiss Merlin FE‐SEM system, and the STEM imaging was captured at room temperature using a Nion UltraSTEM U200 microscope operated at 60 kV.

### Statistics Analysis

All graphene layers employed in this study had a size of about 7 mm × 7 mm, and the GWM has a size of 1 cm × 1 cm. The graphene roughness RMS of the three types of transfer was measured from Veeco AFM Nanoscope software. All MD simulations were carried out by the Large‐scale Atomic/Molecular Massively Parallel Simulator package.^[^ ^23^ ^]^ Graphene with a width of 4 nm and a length of 10 nm was placed on the surface of the liquid mixture of water and IPA with 42 000 water molecules. The simulation box size for peeling was 9.947 nm × 20.4192 nm × 20.0 nm, and the adaptive intermolecular reactive bond order modeled flexible graphene. The SPC/E and TraPPE‐UA potential models^[^ ^24^ ^]^ were adopted for water and IPA molecules, respectively. For the nonbonded interactions, the 12–6 pairwise Lennard–Jones potential *V* (*r*) = 4*ε*(*σ* ^12^/*r* ^12^ − *σ* ^12^/*r* ^12^) and Coulomb interaction *V_q_* (*r*) = *q_i_q_j_*/4*πε*_0_ *r* were applied where *r* is the interatomic distance. At the same time, *σ* and *ε* are the equilibrium distance and the interactive well depth of the potential, respectively, *q_i_* and *q_j_* are the electronic charge counterpart, and *ε*_0_ is the permittivity of the vacuum. Specifically, the Lennard–Jones parameters for graphite‐water interaction were *σ*_*CO*_ = 3.19 Å and *ε*_*CO*_ = 0.00407 *eV*.^[^ ^25^ ^]^ The cut‐off distance was 1 nm in this study. The Lorentz–Berthelot mixing rule was used to determine the inter‐L–J parameters for different components. The particle–particle‐particle–mesh algorithm with a root mean of 0.0001 was used to minimize the error of long‐range Coulombic interactions. All simulations were run in an NVT ensemble with a Nose/Hoover thermostat set at 300 K unless otherwise stated.

## Conflict of Interest

The authors declare no conflict of interest.

## Supporting information

Supporting Information

## Data Availability

The data that support the findings of this study are available from the corresponding author upon reasonable request.
