# Supplementary material for: Discovery of Graphene‐Water Membrane Structure: Toward High‐Quality Graphene Process
Source: Adv Sci (Weinh). 2022 Jul 18;9(26):2201336. doi: 10.1002/advs.202201336 (PMC9475541; doi:10.1002/advs.202201336)
Supplement: Supplementary file 1 — Supporting Information [file ADVS-9-2201336-s001.pdf]

ADVANCED SCIENCE
Open Access

Supporting Information

for *Adv. Sci.*, DOI 10.1002/advs.202201336

Discovery of Graphene-Water Membrane Structure: Toward High-Quality Graphene Process

Aisha Okmi, Xuemei Xiao, Yue Zhang, Rui He, Olugbenga Olunloyo, Sumner B. Harris, Tara Jabegu, Ningxin Li, Diren Maraba, Yasmeen Sherif, Ondrej Dyck, Ivan Vlassiouk, Kai Xiao, Pei Dong, Baoxing Xu* and Sidong Lei**

Supporting information

Discovery of graphene-water membrane structure:

towards high-quality graphene process

Aisha Okmi[1,4#], *Xuemei Xiao*[2#], *Yue Zhang*[2], *Rui He*[3], *Olugbenga Olunloyo*[5], *Sumner B. Harris*[5], *Tara Jabegu*[1], *Ningxin Li*[1], *Diren Maraba*[1], *Yasmeen Sherif*[1], *Ondrej Dyck*[5], *Ivan Vlassiouk*[5] , *Kai Xiao*[5], *Pei Dong*[3]*, *Baoxing Xu*[2]*, *Sidong Lei*[1]*

[1] Department of Physics and Astronomy, Georgia State University, Atlanta, GA 30303, USA.

[2] Department of Mechanical and Aerospace Engineering, University of Virginia, Charlottesville, VA 22904, USA.

[3] Department of Mechanical Engineering, George Mason University, VA 22030, USA

[4]Department of physics, Jazan University, Jazan 45142, Saudi Arabia

[5] Center for Nanophase Materials Sciences (CNMS), Oak Ridge Natioanl Lab, Oak Ridge, TN, 37830

Corresponding Author should be addressed to: slei@gsu.edu; pdong3@gmu.edu; bx4c@virginia.edu

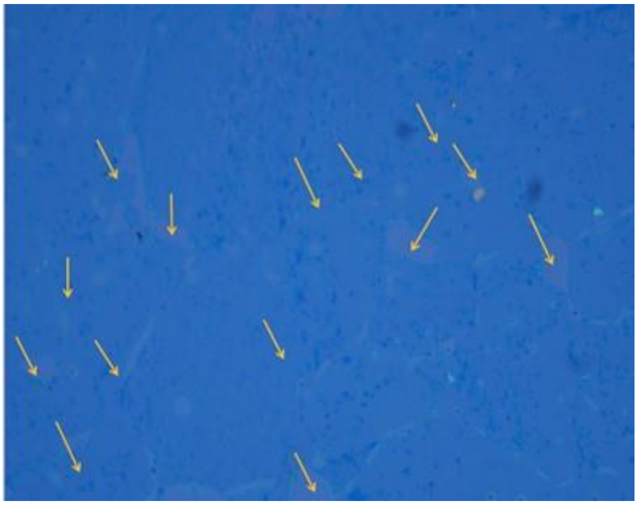

Figure. S1. A density of defects was developed on the graphene layer grown under leakage higher than >10^{-7} bar·cm^3/s.

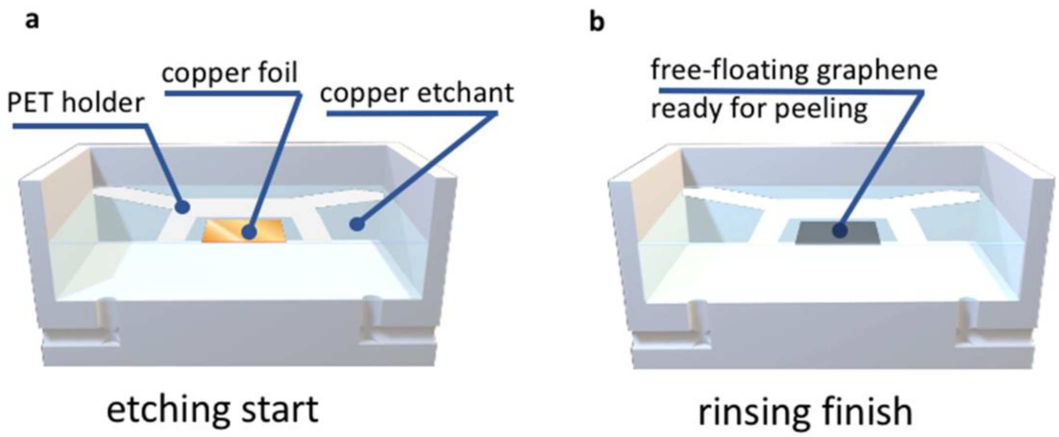

Figure. S2.(a)The copper foil is relocated into a specially designed reactor filled with 0.1 mol/L ammonium persulfate. (b) Graphene floating on pure DI-water that inserted gradually to replace etchant.

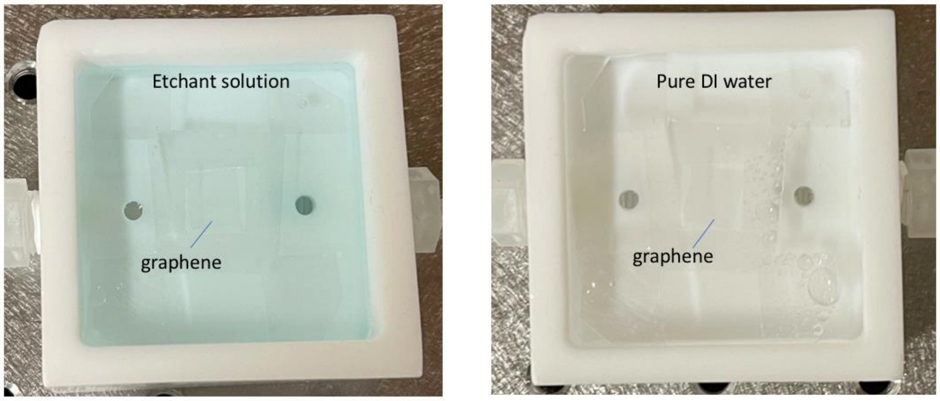

Figure. S3. Free-floating graphene before and after liquids exchange: On the left, graphene floating on 0.1M of ammonium persulfate. On the right, graphene floats on pure DI water.

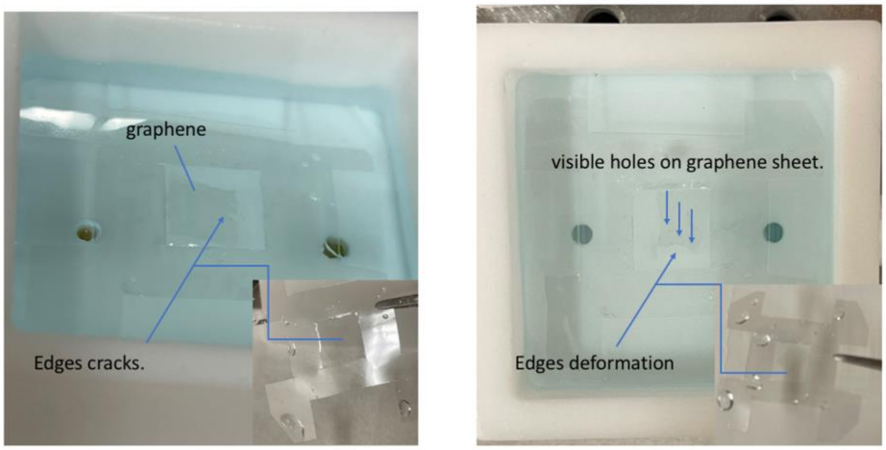

Figure S4. Several defects (edges deformation, shrinking, and visible holes) can be developed during the growth and jeopardize the graphene quality.

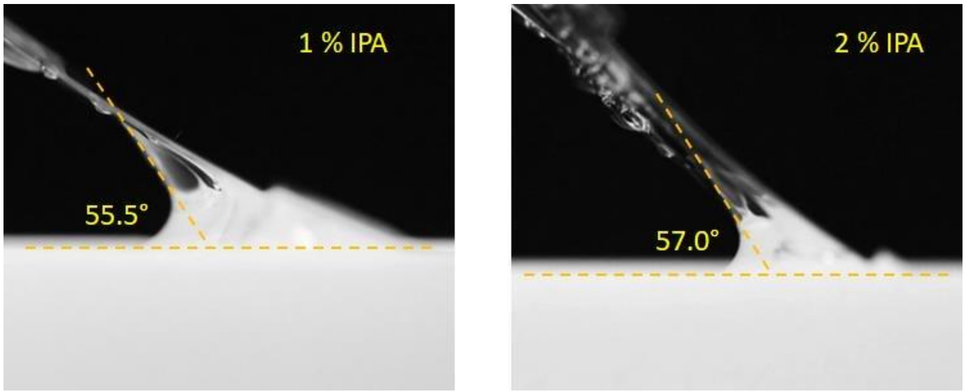

Figure S5. A successful peeling of GWM from a liquid contains 1% IPA to 99% DI water (left), and a liquid contains 2% IPA to 98% DI water (right).

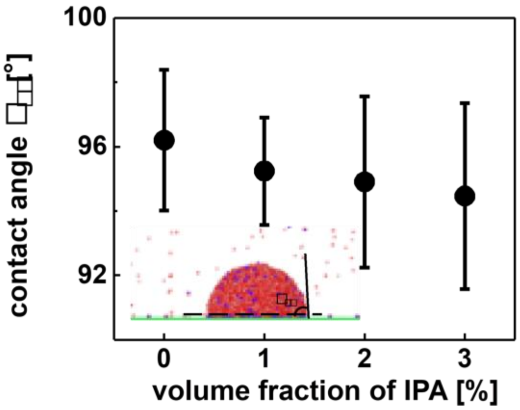

Figure. S6. The contact angle of the IPA-water mixture on graphene was calculated by MD simulations. Error bars indicate standard deviation during the NVE production period.

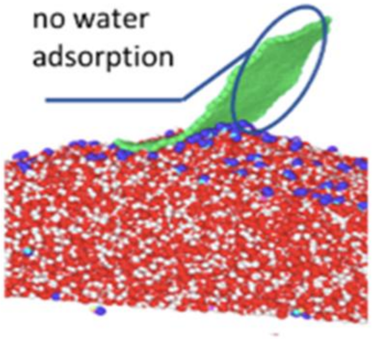

Figure. S7. MD simulation confirms that water molecules do not adhere to the graphene layer being peeled off.

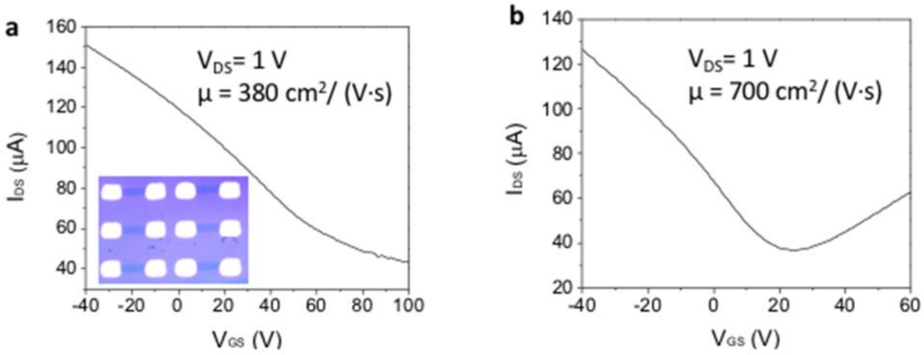

Figure. S8.Field-effect transistors ((a) inset) had been fabricated with the same batch of graphene layers transferred with the conventional and our newly developed polymer-free transfer approach. The FET made with the conventional polymer-free method (a) yields mobility of 380 $cm^2V^{-1}s^{-1}$, whereas our new method renders a significant improvement up to 700 $cm^2V^{-1}s^{-1}$ as shown in (b).
